# Effect of Cooling Rate during Glazing on the Mechanical and Optical Properties of Monolithic Zirconia with 3 mol% Yttria Content

**DOI:** 10.3390/ma14237474

**Published:** 2021-12-06

**Authors:** Mi-Hyang Cho, Hyo-Joung Seol

**Affiliations:** 1 Department of Dental Lab, Wonkwang Health Science University, Iksan-si 54538, Jeonbuk-do, Korea; milgong11@wu.ac.kr; 2 Department of Dental Materials, Dental and Life Science Institute, School of Dentistry, Pusan National University, Yangsan-si 50612, Gyeongsangnam-do, Korea

**Keywords:** zirconia, glazing, cooling rate, mechanical and optical properties, Weibull analysis

## Abstract

Glazing is the final heat treatment process in the manufacturing of a monolithic zirconia prosthesis. Herein, the effect of cooling rate during zirconia glazing was investigated. A 3 mol% yttria-stabilized tetragonal zirconia polycrystal was glazed at the general cooling rate suggested by the manufacturer, as well as at higher and lower cooling rates, and the differences in flexural strength, hardness, optical properties, and crystal structure were evaluated. A higher cooling rate did not affect the flexural strength, hardness, grain size, optical properties, or crystal structure; however, the Weibull modulus decreased by 1.3. A lower cooling rate did not affect the flexural strength, optical properties, or crystal structure; however, the Weibull characteristic strength increased by 26.7 MPa and the Weibull modulus increased by 0.9. The decrease in hardness and the increase in grain size were statistically significant; however, the numerical differences were negligible. This study revealed that a lower cooling rate provides more reliable flexural strength. Therefore, glazing can proceed at a general cooling rate, which takes 3–4 min; however, glazing at a lower cooling rate will provide a more consistent flexural strength if desired, despite being time-consuming.

## 1. Introduction

Zirconia and yttria-stabilized zirconia are tough, hard, strong, and wear- and corrosion-resistant, and they exhibit low coefficients of friction; hence, they are widely used as coating materials in various industries [1,2]. Zirconia is widely used in dentistry for restoration and implants. In addition, titanium implants are coated with zirconia to improve various properties, including their mechanical strength and bioactivity [3,4,5,6]. Zirconia restorations have limitations in satisfying the requirements for esthetics similar to natural teeth, because of the inherent opacity of the material. Therefore, the translucency of natural teeth has been reproduced by fabricating zirconia as the core of the restoration and then veneering dental porcelain. However, this method may cause the porcelain to fracture or fall off because of the low tensile strength of porcelain, and the difference in the coefficients of thermal expansion of porcelain and zirconia [7]. Tensile stress, one of the causes of porcelain fracture, is generated by the bending force during mastication in the oral cavity, and the maximum tensile stress is generated on the surface of the prosthesis. Therefore, surface defects determine the strength of ceramics [8]. A significant difference in the coefficients of thermal expansion of the zirconia core and the veneer porcelain can generate residual stress in the porcelain, which can result in the porcelain being fractured [9,10]. Because of these shortcomings of zirconia–porcelain restorations, zirconia with improved translucency has been manufactured and used in the fabrication of monolithic prostheses.

Porcelain is not veneered when monolithic prostheses are fabricated, which greatly simplifies the fabrication process, and glazing is the only heat treatment required after the full sintering of zirconia. Glazing is typically performed for 1–2 min at a temperature in the range of approximately 850–900 °C. The prosthetic is then cooled to approximately 600 °C and then bench-cooled to room temperature. In the case of porcelain veneered on zirconia, the cooling rate after glazing affects the residual stress within the porcelain [9], and this has been reported as one of the causes of porcelain chipping in porcelain–zirconia restorations. In the case of metal–ceramic restorations, the hardness and microstructure of the metal substructure has been reported to depend on the cooling rate during sintering and glazing after veneering porcelain onto the metal substructure [11,12]. To fabricate monolithic zirconia prostheses, glazing is performed at a temperature somewhat lower than the temperature required for sintering porcelain veneer. Therefore, the temperature range for cooling is relatively narrower in comparison to that for prostheses with porcelain veneer. Consequently, the effects of the cooling rate during glazing on the various properties of zirconia have received little attention.

Pure zirconia has three forms—monoclinic, tetragonal, and cubic structures—under atmospheric pressure, and a phase transformation can occur depending on the temperature. The monoclinic phase is stable at room temperature, and the tetragonal and cubic phases are stable at higher temperatures. The tetragonal phase has an increased toughness resulting from the zirconia undergoing a phase transformation to a monoclinic phase when subjected to stress. A tetragonal or cubic phase can form when a phase stabilizer, such as MgO, CaO, or Y_2_O_3_, is added to pure zirconia, even at room temperature. However, tetragonal zirconia spontaneously undergoes a phase transformation from the tetragonal to monoclinic phase when used at low temperatures for a long time. The resulting volume expansion causes microcracks in the specimen, which result in a rapid decrease in strength, i.e., low-temperature degradation [13]. Changing the composition of zirconia or rapidly cooling a 3–7 mol% yttria-stabilized zirconia after sintering has been reported to generate a metastable tetragonal (t′) phase with an axial ratio closer to 1 than that of a tetragonal phase (t) [14]. Unlike the t phase, the t′ phase is resistant to phase transformation under stress [15,16]; hence, it may be more resistant to low-temperature degradation than the t phase [17].

Numerous studies have been conducted for improving the aesthetics, strength, and toughness of zirconia [18,19,20,21,22,23,24]. Grain boundaries, pores, the presence of secondary phases, and light scattered by rough surfaces have been reported to reduce the translucency of zirconia [25]. The translucency of zirconia can be improved by reducing the Al_2_O_3_ content to less than 0.05% [14,26], adding La_2_O_3_ [14], increasing the Y_2_O_3_ content [27], increasing the sintering temperature [27], and/or reducing the particle size to less than 100 nm, which is less than the wavelength of visible light [28]. Increasing the Y_2_O_3_ content of zirconia is particularly effective in stabilizing the cubic phase. Unlike the tetragonal phase, the cubic phase is optically isotropic, so there is no light scattering due to birefringence, and translucency is remarkably improved. However, unlike tetragonal zirconia, cubic zirconia does not cause a stress-induced phase transformation, so an increase in toughness cannot be expected [14]. Reducing the Al_2_O_3_ content or increasing the La_2_O_3_ or Y_2_O_3_ content to improve transparency also degrades the mechanical properties of zirconia [14,29,30].

Glazing is performed after zirconia sintering during the manufacture of a monolithic zirconia prosthesis. The cooling rate used is typically that suggested by the manufacturer. Cooling from approximately 850 °C to 600 °C usually takes 3–4 min. This is followed by bench-cooling to room temperature. The characteristic changes that occur during the final sintering of zirconia as a function of the cooling rate have been examined in previous studies [30,31,32]. However, the effect of the cooling rate on the properties of zirconia in the glazing step during the fabrication of a monolithic zirconia prosthesis has not yet been reported. Therefore, in this study, the effects of the cooling rate during glazing on the mechanical and optical properties of zirconia were investigated. Uncolored 3 mol% yttria-stabilized tetragonal zirconia polycrystal (3Y-TZP) was subjected to glazing heat treatment at different cooling rates without veneering the glazing solution, and the flexural strength, hardness, microstructure, optical properties, and crystal structure were observed. The following null hypothesis was tested: that the cooling rate during glazing does not affect the mechanical or optical properties, or the crystal structure, of 3Y-TZP.

## 2. Materials and Methods

### 2.1. Specimen Preparation

An uncolored zirconia block (Zenostar T, Wieland Dental, Profzeim, Germany) with an yttria content of 3 mol% (Table 1) was cut in a pre-sintered state with a cutter equipped with a diamond wheel (Accutom-100, Struers Company, Copenhagen, Denmark). Specimens for color measurement were then dry-polished sequentially up to 5000 grit with silicon carbide abrasive paper. All specimens were then sintered at 1450 °C for 2 h in a sintering furnace (Zenotec Fire P1, Wieland Dental, Profzeim, Germany), in accordance with the manufacturer’s instructions. The specimens exhibited sintering shrinkage of approximately 20%, and the final specimen size for flexural strength testing after sintering was 4.0 mm × 1.65 mm × 16.5 mm (±0.04, ISO 6872). The fragments obtained after measuring the flexural strength were used for hardness testing and microstructure observation. The final specimen size for XRD examination was 10 mm × 10 mm × 1.65 mm. The final specimen sizes for color measurement were 10 mm (width) × 10 mm (length) × thicknesses of 0.52 (±0.008), 1.02 (±0.021), 1.53 (±0.011), and 2.02 (±0.011) mm. The size of each specimen was measured with an electronic Vernier caliper (Mitutoyo, Kanagawa, Japan).

### 2.2. Glazing Heat Treatment

Fully sintered zirconia specimens were simulated for glazing without glazing solution in a porcelain furnace (Multimat 2 Torch, Dentsply, Bensheim, Germany), according to the schedule in Table 2. The glazing schedule in Table 2 corresponds to the schedule of the glaze paste for zirconia (CZR-E glaze, Kuraray Noritake, Nagoya, Japan), which is a typical glazing schedule for zirconia. All specimens glazed at 850 °C were cooled down to the starting temperature (600 °C) and then bench-cooled to room temperature. Three cooling rates from 850 °C to 600 °C were used. The group of specimens cooled by immediate removal from the porcelain furnace at 850 °C is denoted C-0 min, the group cooled to 600 °C in 4 min, as suggested by the manufacturer, is denoted C-4 min, and the group cooled to 600 °C in 7 min is denoted C-7 min.

### 2.3. Flexural Strength Testing

Three-point flexural strength testing was conducted using a universal testing machine (Instron 3345, Norwood, MA, USA) according to ISO 6872:2015 (n = 25/group). The distance between the supports was fixed at 12 mm, and the load value when the specimen was fractured by the application of a vertical compression force at a crosshead speed of 0.5 mm/min was measured using PC software (bluhill2, Instron, Norwood, MA, USA). The flexural strength was calculated according to the following formula: σ = 3Nl/2bd^2^, where σ = flexural strength, N = fracture load (N), l = distance between supports (mm), b = width of the specimen (mm), and d = thickness of the specimen (mm).

The variation in flexural strength was characterized by two-parameter Weibull analysis. The Weibull modulus (m) and characteristic strength (σ_0_) were determined based on the median ranking and maximum likelihood method using PC software (Reliability and Maintenance Analyst v5.0.9, engineered software, Chandler, AZ, USA). The Weibull distribution was determined using the following formula:

(1) $$\mathrm{Pf}=1-\exp[-(\frac{\sigma }{\sigma_{0}}){\;}^{m}]$$

where Pf = the fracture probability = (rank − 0.3)/(N + 0.4), N = the number of samples, σ = the flexural strength, σ_0_ = the characteristic strength (the strength corresponding to a probability of failure of 63.2%), and ^m^ = the Weibull modulus.

### 2.4. Hardness Testing

Fragments obtained after flexural strength testing were used for hardness testing. A Vickers hardness value was obtained using a Vickers micro-hardness tester (MVK-H1, Akashi Co., Kawasaki, Japan) with a load of 1 kgf and a dwell time of 10 s (n = 3/group). The hardness was measured eight times for each specimen, and the mean and standard deviation values were calculated.

### 2.5. Field Emission Scanning Electron Microscopy (FE-SEM)

FE-SEM (JSM-7200F, Jeol, Akishima, Japan) was performed using fragments obtained after measuring the flexural strength (n = 3/group). After a specimen was coated with platinum for 90 s, its microstructure was observed with an acceleration voltage of 10 kV. The mean grain size was obtained from the FE-SEM images using Image J software (National Institute of Mental Health, Bethesda, MD, USA). For each group, at least 600 grains were measured.

### 2.6. Optical Property Analysis

Using a computer-controlled spectrophotometer (CM-3600d, Konica Minolta Sensing Inc., Osaka, Japan), the spectral transmittance and reflectance were recorded (n = 3/group) from 360 to 740 nm at 10 nm intervals under CIE (Commission Internationale de l’ Eclairage) standard light sources D65 and 2° (observer). Calibration of the spectrophotometer was performed before each measurement. Each specimen was measured three times. Spectral reflectance data were recorded in reflectance mode by placing the specimen over white (L*: 99.2; a*: −0.03; b*: 0.55) and black backgrounds (L*: 9.45; a*: 0.69; b*: −0.43) in UV embedding mode and specular component excluded (SCE) mode. Spectral transmittance data were recorded in transmittance mode. Light transmittance was analyzed by dividing the overall light transmittance by the overall light transmittance without the specimen in the spectrophotometer to obtain the overall light transmittance as a percentage value. Transmittance is specified as a value between 100% (transparent) and 0% (opaque). The average transmittance (AT), translucency parameter (TP) value, and opalescence parameter (OP) value were obtained as follows. AT is the sum of the transmittance (%) at each wavelength divided by the number of data points [33]. TP is obtained according to the formula TP = [(L^∗^_W_ − L^∗^_B_)^2^ + (a^∗^_W_ − a^∗^_B_)^2^ + (b^∗^_W_ − b^∗^_B_)^2^]^1/2^, where the subscript W indicates color coordinates over a white background and the subscript B indicates color coordinates over a black background [34,35]. OP was calculated according to the formula: OP = [(a^∗^_T_ − a^∗^_R_)^2^ + (b^∗^_T_ − b^∗^_R_)^2^]^1/2^, where the subscript T is transmitted light and the subscript R is reflected light with a black background [34,35]. The relationship between the sample thickness and the AT and TP values in each group was investigated by regression analysis using the exponential function [36] *y* = *a*·exp^(*bx*)^, where *y* corresponds to the AT or TP value, *x* corresponds to the sample thickness, and *a* and *b* correspond to constants.

### 2.7. X-ray Diffractometry (XRD)

The phases in each group were analyzed (n = 1/group) using high-resolution X-ray diffractometry (HRXRD, X’Pert3-powder, PANalytical, EA Almelo, The Netherlands) at 40 kV and 30 mA. The scanning step was 2θ = 0.013°. Cu Kα radiation and a Ni-filter were used.

### 2.8. Statistical Analysis

All test results were analyzed using SPSS 25.0 (Statistical Product and Service Solutions 25.0, IBM Co., Armonk, NY, USA) and a statistical significance level of 0.05. Since the flexural strength values were not normally distributed, statistical significance was determined using the Kruskal–Wallis test. Hardness values were analyzed by means of one-way ANOVA and Tukey HSD tests. Since grain size was not normally distributed, statistical significance was tested using Kruskal–Wallis and Dunn–Bonferroni post hoc tests. The effects of the cooling rate and sample thickness on AT and OP were analyzed by means of two-way ANOVA and Tukey HSD tests. Since TP was not normally distributed, statistical significance was tested using a generalized linear model.

## 3. Results

### 3.1. Flexural Strength

Table 3 shows the results of three-point flexural strength testing and Weibull data analysis of glazed specimens at the three cooling rates considered. The flexural strengths of the test groups were not significantly different (*p* > 0.05). A Weibull analysis was performed to assess the reliability of the flexural strength of each specimen. As the cooling rate decreased, the Weibull modulus increased and, thus, the flexural strength reliability increased. The flexural strength results and Weibull characteristic strength followed similar trends. A comparison of the Weibull characteristic strength values shows that the strength of the C-7 min group (935.9 MPa) is lower than that of the before glazing (Before-G) group, by 13 MPa, but higher than that of the C-0 min and C-4 min groups, by approximately 27 MPa.

### 3.2. Hardness

Table 3 shows hardness with respect to cooling rate during glazing. The C-7 min group exhibited a slightly lower hardness than the C-4 min group (*p* = 0.001). However, no significant differences in hardness values were observed among the C-7 min, the Before-G, and the C-0 min groups.

### 3.3. Microstructure

**Figure 1:**
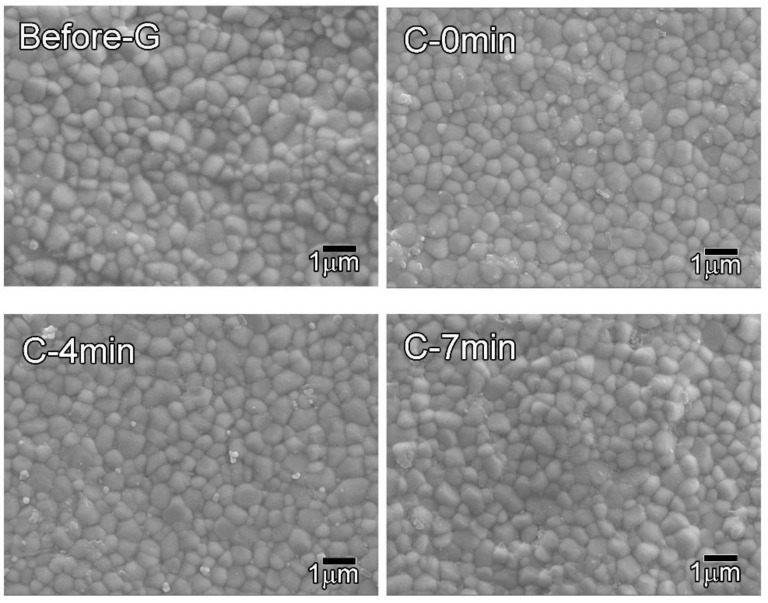
Microstructure of specimens before and after glazing at various cooling rates (×10,000).

Figure 1 shows the microstructure of each group. All groups have similar equiaxed microstructures. The grain size (Table 3) of the C-7 min group was slightly larger than that of the other groups (*p* < 0.001).

### 3.4. Light Transmittance

**Figure 2:**
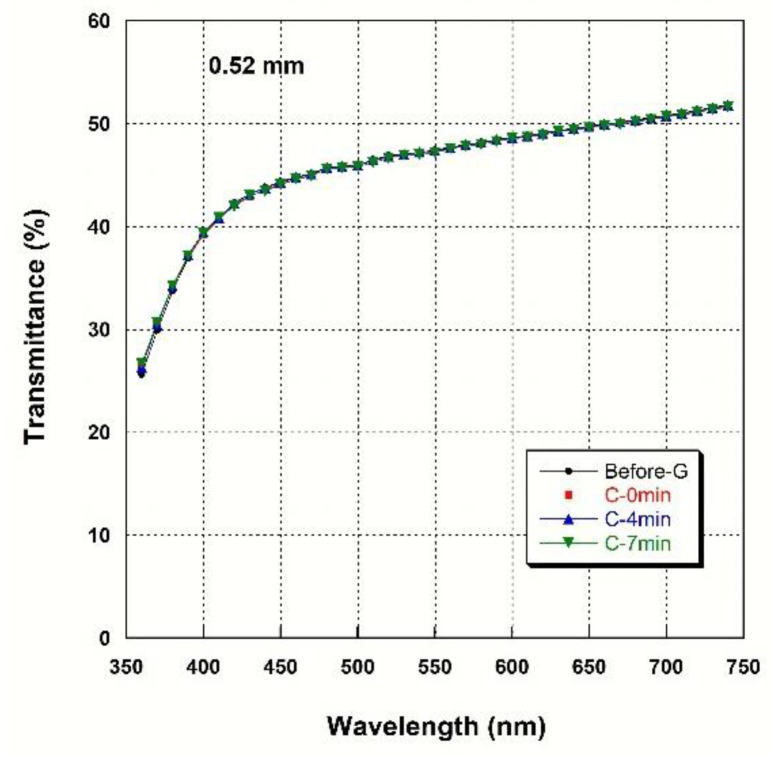
Spectral transmittance curves for various cooling rates at a thickness of 0.52 mm.

**Figure 3:**
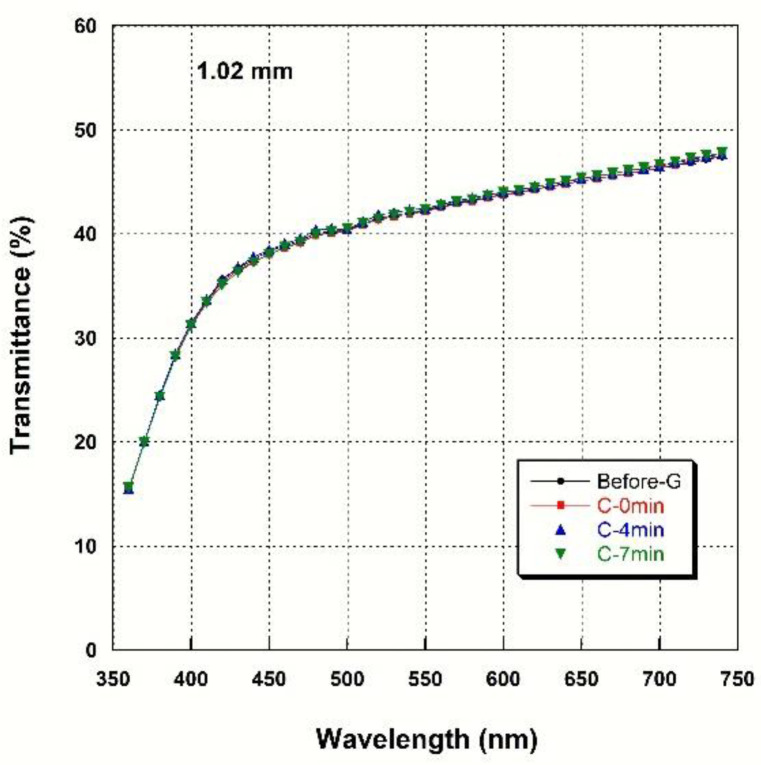
Spectral transmittance curves for various cooling rates at a thickness of 1.02 mm.

**Figure 4:**
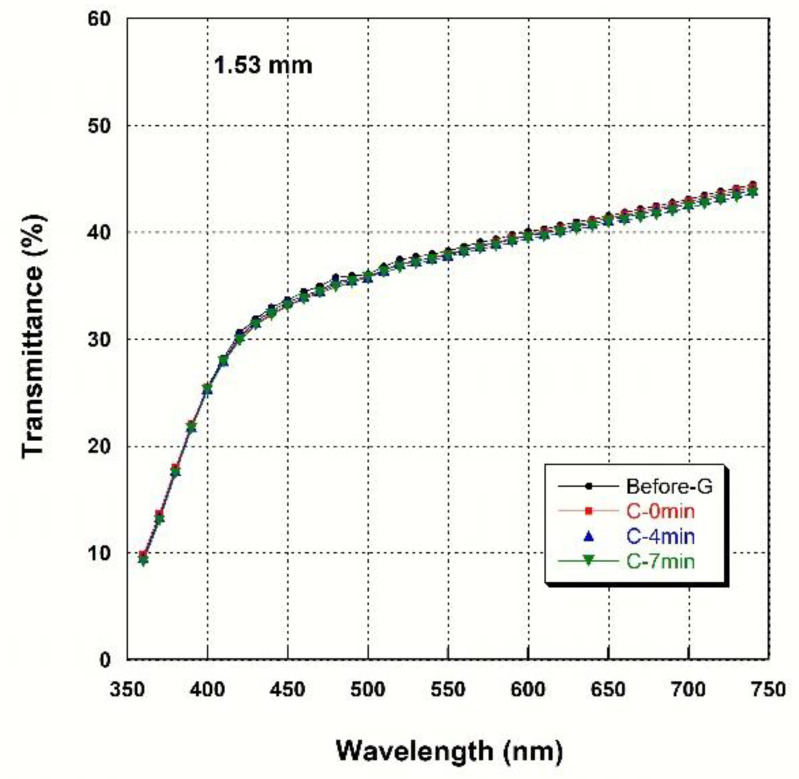
Spectral transmittance curves for various cooling rates at a thickness of 1.53 mm.

**Figure 5:**
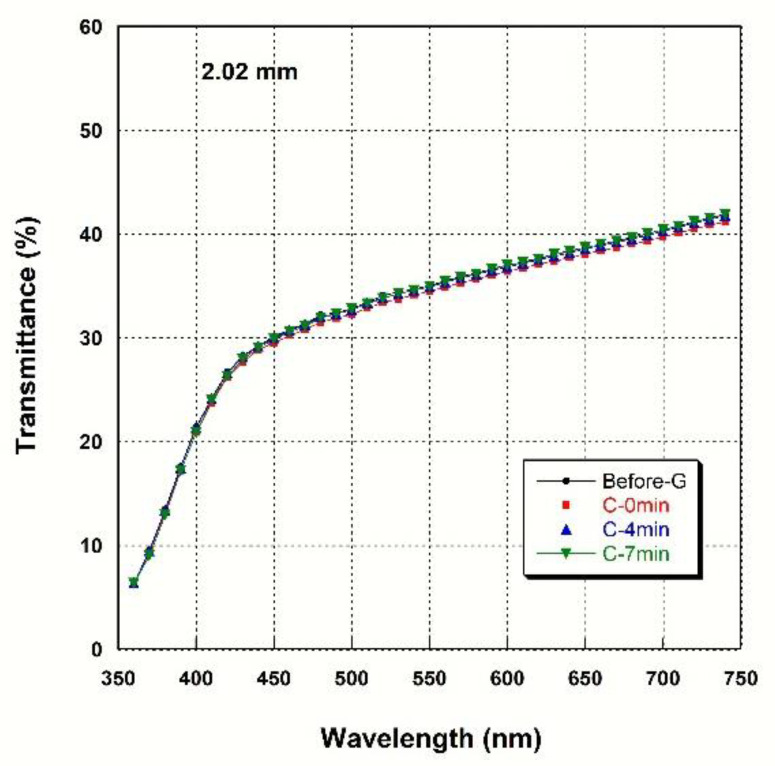
Spectral transmittance curves for various cooling rates at a thickness of 2.02 mm.

Figure 2, Figure 3, Figure 4 and Figure 5 show spectral transmittance according to cooling rate during glazing for specimens of various thicknesses. All groups showed similar spectral transmittance data at various thicknesses. The spectral transmittance values decreased as the thickness increase at all measured wavelengths. In addition, the transmittance gradually decreased as the wavelength decreased for all groups. In particular, the transmittance decreased sharply from 420 nm with decreasing wavelength. Table 4 shows the AT as a function of cooling rate for specimens of various thicknesses. The statistical analysis (Table 4) results show that there is no significant difference in AT according to cooling rate during glazing, and there is no interaction between thickness and cooling rate (*p* > 0.05). Significant differences in AT with thickness were found for all groups. AT decreased from approximately 46% to 32% with increasing thickness (from 0.52 mm to 2.02 mm) (*p* < 0.001).

Regression analysis was performed to assess the relationship between thickness and AT, and the calculated regression equation and correlation coefficients (R^2^) are shown in Table 5. In the regression equations shown, *x* is the sample thickness and *y* is the AT. The constants of the regression equation obtained in each group are similar. Since the correlation coefficients obtained for all groups are very high, we conclude that AT decreases exponentially with increasing thickness.

### 3.5. Translucency Parameter (TP)

Table 6 shows TP as a function of cooling rate for specimens with various thicknesses. There were no significant differences in the TP values for all groups at thicknesses of 1.02, 1.53, and 2.02 mm. The TP was higher by 0.33 in the C-4 min group (*p* = 0.011) and by 0.35 in the C-7 min group (*p* = 0.008), compared to that in the Before-G group, at a thickness of 0.52 mm. Therefore, TP was only higher at a 0.5 mm thickness as a result of glazing at the cooling rates of C-4 min and C-7 min. Significant differences in TP with thickness were evident for all groups, and TP decreased with increasing thickness (*p* < 0.001). There was no interaction between thickness and cooling rate (*p* > 0.05).

Regression analysis was used to assess the relationship between TP and thickness, and the calculated regression equation and correlation coefficients (R^2^) are shown in Table 7, where *x* is the thickness of the sample and *y* is TP. The *a* and *b* values of the regression equation obtained for each group are similar. Since the correlation coefficients obtained for all groups are very high, we conclude that TP decreases exponentially with increasing thickness.

### 3.6. Opalescence Parameter (OP)

Table 8 shows OP as a function of cooling rate for specimens with various thicknesses. There was no significant difference in OP in all groups, and there was no interaction between thickness and cooling rate (*p* > 0.05). OP increased from approximately 6 to approximately 12 as the thickness increased from 0.52 mm to 2.02 mm in all groups (*p* < 0.001). OP did not change exponentially with thickness; instead, OP increased parabolically with increasing thickness.

### 3.7. XRD Study

**Figure 6:**
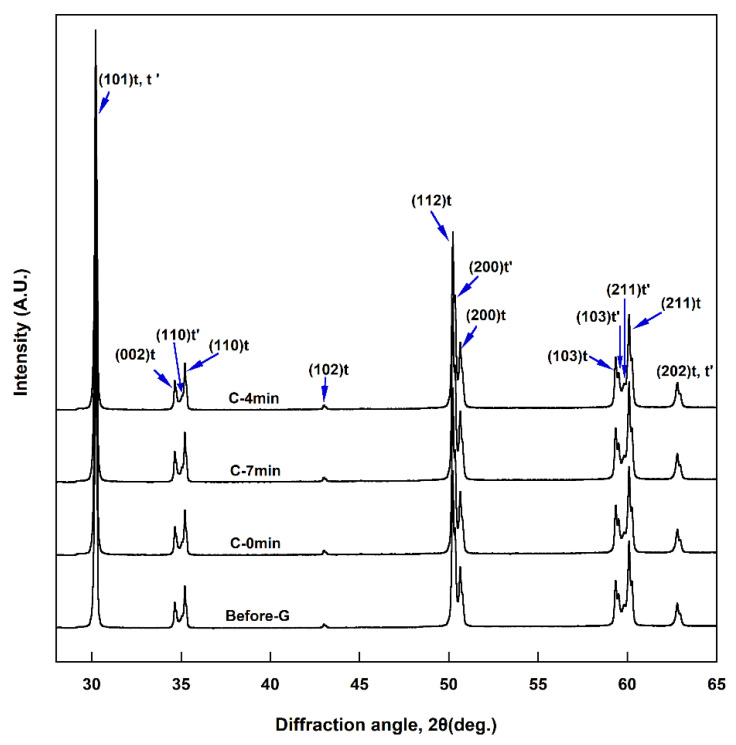
XRD patterns of specimens before and after glazing at various cooling rates.

Figure 6 shows the XRD pattern of each specimen. The lattice constant and Miller index of tetragonal zirconia can be described by a face-centered tetragonal lattice, but in this study, it was described as a more general body-centered tetragonal lattice [37]. No cubic phase or monoclinic phase was observed in any of the specimens; only the tetragonal (t) phase was observed. The metastable tetragonal (t′) phase, which has an axial ratio (*c/a* ratio = c/√2a) closer to one, was also observed to coexists with the t phase. The crystal structure was not observed to change with cooling rate during glazing. The lattice constants of the t phase determined in this study are a = 3.602 Å and c = 5.172 Å, and the lattice constants of the t′ phase are a = 3.624 Å and c = 5.159 Å.

## 4. Discussion

In this study, 3Y-TZP was glazed by heat treatment at various cooling rates after sintering, and the mechanical and optical properties as well as the crystal structure were investigated. The cooling rate during glazing was set higher (C-0 min) and lower (C-7 min) in addition to the cooling rate suggested by the manufacturer (C-4 min), as shown in Table 2. The null hypothesis, i.e., that the cooling rate during glazing does not affect the mechanical and optical properties and crystal structure of 3Y-TZP, was only partially rejected. The flexural strength after glazing was lower compared to that before glazing [38], and the average flexural strength increased with a decreasing cooling rate. However, statistically significant differences were not observed among the test groups (*p* > 0.05).

In general, predicting probable strengths for brittle materials such as ceramics is difficult, and variations in strength are large. Therefore, comparing the mechanical properties of these materials using a Weibull distribution rather than simply comparing the average strengths and standard deviations is useful [39]. The larger the slope of the dispersion of the data (that is, the larger the Weibull modulus (m) value), the higher the reliability of the strength of the specimen. As a result of performing Weibull analysis to confirm the reliability of the flexural strength of each specimen, the C-7 min group was found to have the highest Weibull modulus (9.4) among the groups, indicating that the group with the lowest cooling rate has the most reliable strength. The Weibull characteristic strength (σ_0_) corresponds to the stress when the material’s probability of failure is 63.2%. The C-7 min group exhibited a greater Weibull characteristic strength (935.9 MPa) than the C-0 min and C-4 min groups. The C-0 min group with the highest cooling rate exhibited the lowest reliability, with a Weibull modulus of 7.2. Kim et al. [30] reported that flexural strength was significantly reduced as a result of rapid air-cooling within 1–2 min after sintering 3Y-TZP, which was attributed to the occurrence of subcritical crack propagation. In this study as well, there is the possibility that subcritical crack propagation may have occurred as a result of the induction of residual stress caused by the rapid temperature change in the specimens cooled at a higher cooling rate (C-0 min). The hardness values of all test groups were within the range of 1308–1339 HV, with the C-7 min group having slightly lower values than the C-4 min group. These hardness values correspond to the values reported in the literature for 3Y-TZP [40].

The results of microstructural observation show that the grain size of the C-7 min group was slightly larger than that of the other groups (*p* < 0.001). This means that the grains grew slightly due to slow cooling during glazing. In general, grain size growth is known to have a detrimental effect on the strength of zirconia [41]. In this study, since the grain size growth in the C-7 min group was not severe, it does not appear to have had a detrimental effect on flexural strength. Sen and Isler [40] sintered uncolored highly translucent 3Y-TZP (VITA YZ HT), super translucent 5Y-TZP (VITA YZ ST), and extra translucent 6Y-TZP (VITA YZ XT) [40], and obtained grain sizes of 0.437, 0.729, and 0.815 µm, respectively, with increasing yttria content. The grain size that they obtained for highly translucent 3Y-TZP is similar to that obtained in this study (0.45–0.51 µm), and with increasing yttria content, the tetragonal phase decreased as the cubic phase increased, which improved translucency [40].

In this study, spectral transmittance and spectral reflectance were recorded to evaluate the optical properties as a function of the cooling rate during glazing, and AT, TP, and OP were determined. There was no significant difference in AT for different cooling rates (*p* > 0.05). AT decreased as the thickness increased in all groups, regardless of the cooling rate (*p* < 0.001). Such a decrease in transmittance with thickness has also been reported for colored 3Y-TZP [42]. Regression analyses of the relationships between AT and thickness led to very similar regression equation constants for each group. The high correlation coefficients for all groups indicate that AT decreased exponentially with thickness in all groups. Light transmittance (T) in a homogeneous material is known to follow an exponential function of thickness (T = exp^(-αx)^) [43]. In this equation, a larger α (linear attenuation coefficient) value corresponds to a more opaque material, because attenuation occurs as a result of the scattering or absorption of light in materials, and the degree of scattering and/or absorption of light increase as α increases [43]. In the present study, polycrystals rather than homogeneous materials were used, but the AT of light followed an exponential function similar to that for a homogeneous material [33]. Shiraishi and Watanabe analyzed the AT of a Ce-TZP/Al_2_O_3_ nanocomposite (NANOZR) used as the core material in an all-ceramic prosthesis [33]. They found that the AT was 2% at a thickness of 0.51 mm, which is much lower than the values obtained in this study for 0.52 mm-thick specimens (45.72–45.84%). In the regression equation (*y* = *a*·exp^(*bx*)^) obtained as a result of the regression analysis of the relationship between AT and thickness in NANOZR, the absolute value of *b* that corresponds to the α value in a homogeneous material was 4.267 [33]. The values obtained in this study were much lower, in the range of 0.225–0.237. This demonstrates that light scattering and/or absorption of the 3Y-TZP used in this study are smaller than that of NANOZR. The apparently low AT value of NANOZR was reported to be caused by significant light scattering at the phase boundaries of two phases (Ce-TZP and Al_2_O_3_) with a large difference in refractive index [33].

Translucency is the property of a material whereby most of the transmitted light is scattered, while transparency is the property whereby a negligible portion of the transmitted light undergoes scattering [44]. An examination of the change in TP of each group shows that a significant difference in TP was only observed for a thickness of 0.52 mm. That is, TP was higher with glazing in the C-4 min and C-7 min groups compared to the group before glazing. TP decreased with increasing thickness in all groups (*p* < 0.001). Regression analysis of the relationship between TP and thickness indicates that TP decreased exponentially with increasing thickness, as was observed for AT [33]. Wang et al. [36] investigated changes in translucency with thickness for several types of dental ceramics, and found that in the case of a ceramic with high translucency, the increase in TP with decreasing thickness was larger than that of a ceramic with less translucency. Elsaka [45] analyzed TP using the same zirconia as in this study, and found it to be 15.88 when the thickness was 1.0 mm before sintering. As the specimens were cut 20% larger to compensate for shrinkage [35], the final thickness may have been approximately 0.8 mm. Thus, the reported TP value (15.88) corresponds to a value between 17.24 and 12.11, which are the values obtained in this study for thicknesses of 0.52 mm and 1.03 mm, respectively. Increasing the yttria content in yttria-stabilized zirconia is known to increase the cubic phase content, which greatly increases translucency, thereby improving the esthetics [16,40]. The zirconia used in this study was 3 mol% yttria-stabilized zirconia, and its TP was lower than that of 4 mol% and 5 mol% yttria-stabilized zirconia [40].

Opalescence, derived from the name of the opal stone, is the process by which a material seems yellow in transmitted light and blue in scattered light perpendicular to the transmitted light [44]. Opalescence is believed to increase natural tooth-like vitality in restorations. Analysis of OP versus the cooling rate during glazing shows that there was no significant difference in OP (*p* > 0.05). OP increased from approximately 6 to 12 with increasing thickness (from 0.52 mm to 2.02 mm) in all groups (*p* < 0.001). The change in OP with thickness did not follow an exponential function; instead, OP increased parabolically. Alghazzawi [46] reported an OP value of 6.1 for a 0.4 mm-thick light-colored Zenostar T block, which is similar to the OP value obtained in this study (6.2) for an uncolored block of a slightly greater thickness (0.52 mm). Alghazzawi [46] also found that zirconia blocks from several manufacturers that were pre-colored with A2 shade or treated with A2 coloring liquid exhibited higher OP values (in the range of 7.5–12.4) when the thickness was 0.4 mm.

The XRD results show that the crystal structure was unaffected by cooling rate during glazing. No cubic phase or monoclinic phase was observed in any group; only the tetragonal phase was observed. In particular, the t′ phase, which has an axial ratio (*c/a* ratio = c/√2a) closer to one than the t phase, coexisted with the t phase. Since the t′ phase has a higher yttria content than the t phase, the axial ratio is slightly lower [7]. In this study, the axial ratio of the t′ phase (1.0066) was slightly lower than the axial ratio of the t phase (1.0153). Compared to the t phase, the t′ phase had a lattice constant a little closer to cubic, so the scattering of light due to birefringence was reduced, leading to increased translucency [30]. Kim reported that the t′ phase was increased by a rapid cooling protocol during the sintering of monolithic zirconia containing 3–5 mol% Y_2_O_3_, which improved the translucency [30]. In Kim’s study, the lattice constant and axial ratio of the t-phase (a = 3.6045 Å, c = 5.1787, *c/a* ratio = 1.0159) and t′ phase (a = 3.6218 Å, c = 5.1533, *c/a* ratio = 1.0061) were very similar to those obtained in this study [30].

Based on the experimental results obtained for 3Y-TZP, we conclude that the cooling rate during glazing affects the hardness and grain size, but does not affect the flexural strength, light transmittance, opalescence parameter, translucency parameter, or crystal structure. However, the Weibull modulus increases as the cooling rate decreases and, thus, the reliability of the flexural strength increases. The group of specimens cooled at the lowest rate showed greater Weibull characteristic strengths than the groups cooled at the recommended rate or higher. Therefore, within the limitations of this study, we conclude that glazing at a lower cooling rate will provide a more consistent flexural strength if desired, despite being time-consuming. A limitation of this study is that only one type of 3Y-TZP zirconia was used, and only uncolored zirconia was exposed to a range of cooling rates during glazing. Further studies are needed on the mechanical and optical properties of zirconia of various compositions, especially with Al_2_O_3_ contents known to decrease transparency but improve the mechanical properties of zirconia, as well as yttria and other important components.

## 5. Conclusions

Within the range of conditions considered in this study, the use of a lower cooling rate than that suggested by the manufacturer during glazing did not affect the flexural strength, optical properties, or crystal structure of 3Y-TZP. However, the Weibull characteristic strength obtained from the flexural strength increased by 26.7 MPa, and the Weibull modulus increased by 0.9. In addition, the hardness decreased slightly while grain size increased slightly (*p* < 0.05), but the numerical difference was negligible. The use of a higher cooling rate than that suggested by the manufacturer did not affect flexural strength, hardness, grain size, optical properties, or crystal structure; however, the Weibull modulus decreased by 1.3, according to Weibull analysis results. AT decreased exponentially, from approximately 46% to 32%, with increasing thickness (0.52 mm to 2.02 mm) in all groups (*p* < 0.001), while TP decreased exponentially, from approximately 17.5 to approximately 6 (*p* < 0.001), and OP increased from approximately 6 to approximately 12 (*p* < 0.001).

## Figures and Tables

**Table 1:** Chemical composition of the pre-sintered zirconia block.

Elements	ZrO_2_ + HfO_2_ + Y_2_O_3_	Y_2_O_3_	HfO_2_	Al_2_O_3_ + Other Oxides
Content (wt.%)	≥99.0	>4.5–≤6.0	≤5	≤1.0

**Table 2:** Simulated glazing process.

Pre- Drying (min)	Heat Rate (°C/min)	Start Temp. (°C)	Final Temp. (°C)	Hold Time (min)	Vacuum Level (cm/HG)	Start Vacuum (°C)	Vacuum Release (°C)	Cooling Rate (°C/min)
5	65	600	850	1	72	600	850	>250 (C-0 min) 62.5 (C-4 min) 35.7 (C-7 min)

**Table 3:** Flexural strength, Weibull modulus, characteristic strength, hardness, and mean grain size in each group.

Code	Sample Number	Mean Flexural Strength ± SD (MPa)	Weibull Modulus (m) (95% Confidence Interval)	Characteristic Strength, σ_0_ (MPa) (95% Confidence Interval)	Mean, HV (±SD)	Mean Grain Size ±SD (μm)
Before-G	25	904.0 ^a^ (99.9)	9.3 (7.0–12.3)	949.0 (907.2–992.7)	1325.7 ^ab^ (36.6)	0.47 ^a^ (0.17)
C-0 min	25	848.9 ^a^ (149.2)	7.2 (5.2–9.9)	909.0 (858.2–962.7)	1327.6 ^ab^ (26.3)	0.45 ^a^ (0.17)
C-4 min	25	858.0 ^a^ (128.1)	8.5 (6.2–11.5)	909.2 (866.0–955.0)	1338.9 ^b^ (23.0)	0.47 ^a^ (0.19)
C-7 min	25	887.0 ^a^ (114.2)	9.4 (6.8–13.0)	935.9 (895.5–978.1)	1308.1 ^a^ (30.7)	0.51 ^b^ (0.17)

The same superscript letter in the same column indicates no statistically significant difference (*p* > 0.05).

**Table 4:** AT as a function of cooling rate for specimens with various thicknesses (mean ± SD).

Thickness (mm)	Before-G	C-0 min	C-4 min	C-7 min
0.52	45.82 ^D,a^ (0.32)	45.78 ^D,a^ (0.17)	45.84 ^D,a^ (0.04)	45.72 ^D,a^ (0.41)
1.02	40.10 ^C,a^ (0.59)	40.04 ^C,a^ (0.21)	40.30 ^C,a^ (0.18)	40.16 ^C,a^ (0.35)
1.53	35.88 ^B,a^ (0.33)	35.61 ^B,a^ (0.56)	35.54 ^B,a^ (0.56)	35.26 ^B,a^ (0.83)
2.02	32.66 ^A,a^ (0.49)	32.09 ^A,a^ (0.72)	32.53 ^A,a^ (0.35)	32.50 ^A,a^ (0.51)

The same uppercase letter indicates no statistically significant difference among thicknesses (*p* > 0.05), and the same lowercase letter indicates no statistically significant difference among groups (*p* > 0.05).

**Table 5:** Regression analysis of the relationship between AT and thickness in each group.

Code	Regression Equation	R^2^	P
Before-G	*y* = 50.774exp^−0.225*x*^	0.988	<0.001
C-0 min	*y* =51.145exp^−0.237*x*^	0.989	<0.001
C-4 min	*y* =51.024exp^−0.231*x*^	0.989	<0.001
C-7 min	*y* =50.826exp^−0.231*x*^	0.980	<0.001

**Table 6:** TP as a function of cooling rate for specimens with various thicknesses (mean ± SD).

Thickness (mm)	Before-G	C-0 min	C-4 min	C-7 min
0.52	17.24 ^D,a^ (0.20)	17.48 ^D,ab^ (0.18)	17.57 ^D,b^ (0.08)	17.59 ^D,b^ (0.05)
1.02	12.11 ^C,a^ (0.47)	12.25 ^C,a^ (0.19)	12.15 ^C,a^ (0.36)	12.26 ^C,a^ (0.14)
1.53	8.70 ^B,a^ (0.26)	8.72 ^B,a^ (0.05)	8.86 ^B,a^ (0.10)	8.74 ^B,a^ (0.06)
2.02	6.27 ^A,a^ (0.07)	6.07 ^A,a^ (0.26)	6.11 ^A,a^ (0.10)	6.16 ^A,a^ (0.05)

The same uppercase letter indicates that there is no statistical differences among thickness (*p* > 0.05), and the same lowercase letter indicates no statistical differences among groups (*p* > 0.05).

**Table 7:** Regression analysis of the relationship between TP and thickness in each group.

Code	Regression Equation	R^2^	P
Before-G	*y* = 23.936exp^−0.673*x*^	0.996	<0.001
C-0 min	*y* = 24.844exp^−0.703*x*^	0.997	<0.001
C-4 min	*y* = 24.771exp^−0.697*x*^	0.998	<0.001
C-7 min	*y* = 24.803exp^−0.697*x*^	1.0	<0.001

**Table 8:** OP as a function of cooling rate for specimens with various thicknesses (mean ± SD).

Thickness (mm)	Before-G	C-0 min	C-4 min	C-7 min
0.52	6.20 ^A,a^ (0.16)	6.42 ^A,a^ (0.13)	6.33 ^A,a^ (0.27)	6.39 ^A,a^ (0.36)
1.02	8.89 ^B,a^ (0.21)	8.94 ^B,a^ (0.05)	8.74 ^B,a^ (0.19)	9.01 ^B,a^ (0.14)
1.53	10.77 ^C,a^ (0.22)	10.83 ^C,a^ (0.11)	10.74 ^C,a^ (0.27)	10.97 ^C,a^ (0.12)
2.02	12.31 ^D,a^ (0.10)	11.69 ^D,a^ (0.61)	11.93 ^D,a^ (0.22)	12.17 ^D,a^ (0.17)

The same uppercase letter indicates that there is no statistical differences among thickness (*p* > 0.05), and the same lowercase letter indicates no statistical differences among groups (*p* > 0.05).

## Data Availability

Not applicable.
